# Trends and predictors of mother-to-child transmission of HIV in an era of protocol changes: Findings from two large health facilities in North East Nigeria

**DOI:** 10.1371/journal.pone.0224670

**Published:** 2019-11-11

**Authors:** Ademola Joshua Itiola, Ameena Ebrahim Goga, Vundli Ramokolo

**Affiliations:** 1 School of Public Health, University of the Western Cape, Bellville, Cape Town, South Africa; 2 Health Systems Research Unit, South African Medical Research Council, Pretoria, South Africa; 3 HIV Prevention Research Unit, South African Medical Research Council, Pretoria, South Africa; 4 Department of Paediatrics, University of Pretoria, Pretoria, South Africa; University of Cincinnati College of Medicine, UNITED STATES

## Abstract

**Background:**

Research studies have demonstrated a reduction in the risk of mother-to-child transmission of HIV (MTCT) to less than 2%, or 5% in non-breastfeeding and breastfeeding populations, respectively, with antiretroviral interventions. However, the risk of MTCT in routine health-facility settings, where service delivery is usually sub-optimal needs monitoring.

**Method:**

We conducted a retrospective review of data from 2008–2014, in two health facilities in Adamawa State, Nigeria. Descriptive statistics were used to estimate overall MTCT rate and MTCT rate by year, and period of prevention of mother-to-child transmission of HIV (PMTCT) protocol implementation. We conducted simple and multiple logistic-regression analyses, to identify predictors of MTCT.

**Results:**

Data from 1,651 mother-to-infant pairs, with HIV deoxyribonucleic acid (DNA) polymerase-chain reaction (PCR) test results from 2008 (n = 49), 2009 (n = 246), 2010 (n = 280), 2011 (n = 335), 2012 (n = 290), 2013 (n = 225) and 2014 (n = 226) were analysed. The overall MTCT rate among HIV exposed infants (HEIs) was 9.7% (95% CI 8.3% - 11.1%) at a median age of 8 weeks (IQR = 6–20). The MTCT rate decreased from 14.3% (4.4%-24.2%) in 2008 to 4.9% (2.1%-7.7%) in 2014 (p = 0.016). The MTCT rate was the lowest (5.4% [3.7% - 7.0%]) when all pregnant women living with HIV received triple antiretroviral therapy, as treatment or prophylaxis (ARVT/P). Using the pooled data, we found that infant age, breastfeeding option, antiretroviral regimen and year were predictors of MTCT. The adjusted odds of MTCT were significantly higher, when neither mother nor HEI received ARVT/P (Adjusted odds ratio (AOR) 26.4 [14.0–49.8], and lower amongst infants born in 2012, compared with those born in 2008 (AOR 0.2 [0.0–1.0]).

**Conclusion:**

The MTCT rate declined significantly between 2008 and 2014 in these two routine health-facility settings in Nigeria. Our study suggests that ARVT/P yields the lowest MTCT. Thus, efforts to scale up lifelong ARVT/P (Option B+) in Nigeria should be accelerated.

## Introduction

By 2015, Nigeria had the highest number of children living with HIV globally, estimated at 260 000 [1, 2]. Almost a fifth of these children (41 000) were newly infected with HIV in 2015, representing 27% of the global new pediatric HIV infections for the year [1]. Approximately 2.2 million children in Nigeria have been orphaned by HIV/AIDS [3]. To prevent mother-to-child transmission of HIV (MTCT), Nigeria implemented the prevention of mother-to-child transmission of HIV (PMTCT) programme in 2002, in six health facilities [4]. By 2014, the programme had been scaled up to 6000 facilities, which represents approximately 18% of the health facilities in Nigeria [5, 6, 7]. The adult HIV prevalence in Nigeria is estimated to be 1.4%, as of 2018, with an antenatal HIV prevalence of 2.9% in 2014 [8, 9].

As recommended by the World Health Organization (WHO), Nigeria implements a comprehensive 4-pronged strategy to prevent HIV/AIDS in infants and children [3,10]. The first PMTCT guideline in Nigeria was produced in 2001; and it was updated in 2005, 2007, 2010, 2014 and 2016 [4,11]. Both the eligibility criteria and the recommended antiretroviral drugs (ARVs) changed with each revision–from zidovudine during pregnancy–to triple ARV therapy during pregnancy, as treatment or prophylaxis (ARVT/P) by 2010 to ART during pregnancy by 2014, S1 Table [3,11,12].

Despite the implementation of the PMTCT programme in Adamawa State, Nigeria; since September 2007, no study has been conducted to investigate PMTCT effectiveness. To date, the only country-level findings available in Nigeria are from operational research conducted in 2005, when only 11 tertiary sites offered PMTCT services [4]. Most of the facility-based PMTCT effectiveness studies in Nigeria were single-site studies conducted at tertiary or teaching hospitals [13–18]. Most published Nigerian studies also reported findings during one period of the PMTCT protocol [13,14,19–24]. Although a review by Khamofu et al., (2015) reported MTCT rates from 2008–2014. This study did not aggregate MTCT rates by PMTCT protocol period [6]. Additionally, the study did not report age of testing, confidence intervals of MTCT rates or predictors of MTCT [6].

We, therefore, conducted a study to evaluate PMTCT effectiveness in two health facilities in Adamawa State, Nigeria over a 7-year period (2008–2014) of PMTCT programme implementation. This represents two periods of PMTCT protocol changes (2007 and 2010), as well as a transitional period, during which both the 2007 and the 2010 PMTCT guidelines were implemented. Additionally, we report predictors of MTCT using the pooled data, controlling for year.

## Methodology

We conducted a retrospective review of routine facility-held records; and analyzed routine individual-level patient data extracted from facility-based registers, from January 2008 to December 2014 (S1 File). All data were analyzed cross-sectionally. Records reviewed included those from early-infant diagnosis (EID)/Infant follow-up registers and HIV PCR request/result forms accessed from the PMTCT and medical record units of the hospitals. The review was conducted at Specialist Hospital (SH) Yola, a health facility providing predominantly secondary healthcare services in the Yola North Local Government Area (LGA), and Federal Medical Centre (FMC) Yola, a tertiary health facility in the Yola South LGA. The two health facilities commenced delivery of PMTCT services in September 2007 and January 2008, respectively. Other services rendered by both of these facilities included antenatal care (ANC), general out-patient and specialist services.

The facilities render services to pregnant women and HIV-exposed infants (HEIs), in accordance with national cascades and protocols (S1 Fig and S1 Table). Using Lasec® DBS Collection kits with 5 spots, infant dried blood spot (DBS) samples were collected and couriered to the Federal Medical Centre, Jalingo. This centralized HIV deoxyribonucleic acid polymerase-chain reaction (DNA PCR) testing facility uses the Roche® brand of PCR machine; and is located approximately 167 kilometres away from Yola.

### Definitions

We defined MTCT rate as the proportion of tested HIV-exposed infants (HEIs) who tested HIV positive. This formed the basis of our evaluation of PMTCT effectiveness as it allowed us to assess MTCT reduction in routine health-facility settings.

For the overall MTCT rate, the MTCT rate by year, and the MTCT rate by PMTCT protocol period, the denominators were the total number of HEIs tested for HIV, with DNA PCR results: for the entire seven-year period, during a specific year, and during the specific protocol period; while the numerators were the number of HEIs that tested HIV positive: for the entire seven-year period, during a specific year, and during the specific period.

We defined Period 1, as the period when only the 2007 National Guidelines were used (Jan 2008 –Jan 2010), and Period 2 as the period when only the 2010 National Guidelines were used (Jun 2012-Dec 2014). The transitional period refers to the period when both the 2007 and the 2010 Guidelines were used (Feb 2010 –May 2012). We reviewed regimens taken by HIV- positive pregnant women and HEIs, in order to confirm the duration of the transition period. From the pooled data, we estimated the transitional period to be two years and four months (Feb 2010 –May 2012).

We defined exclusive breastfeeding as feeding an infant with breast milk only [25]. This excluded the use of formula feed, or any other liquids or solids [25]. The use of the prescribed medications and oral rehydration salt (ORS) for diarrhea was, allowed, as per WHO definitions [25]. We defined mixed feeding as feeding an infant with both breast milk and formula feed, or any other liquid, or solids [25]. Lastly, replacement feeding/not breastfed at all, refers to avoiding all breastmilk and feeding an infant with an appropriate replacement milk [25].

### Data analysis

The data were analyzed by using STATA 14.2 [26]. Descriptive statistics (proportions) were used to describe the overall MTCT rate, MTCT rate by year, and the PMTCT protocol periods. Simple and multiple logistic regression analyses were conducted to establish the predictors of MTCT. Covariates were selected, based on the current literature on the risk factors for MTCT [19,21, 23, 24, 27–29]. Specifically, we included gender, infant age, breastfeeding option, ART/ARV prophylaxis receipt, hospital and year, in our logistics regression model. A p-value <0.05 was considered statistically significant. No adjustment was made in estimating the confidence intervals.

### Data management

All 158 infants, without first HIV DNA PCR test results, were excluded from all analyses. HIV-exposed infants excluded from the analyses differed significantly by age and feeding option, as well as by infant and maternal ARV use, compared with HEIs included in the analyses (S2 Table). For longitudinally linked results, if an infant tested HIV negative at first test and HIV positive at second testing, the HIV positive result was used in the analysis.

### Ethics approval

Ethics approval for the study was obtained from the Senate Research Committee of the University of the Western Cape. Permission to access patient records was obtained from the research ethics committees of the two health facilities involved in this study, and from the Adamawa State Ministry of Health (SMoH). No individual patient-level consent was obtained; as no patient was interviewed. All data were de-identified: names of patients were not extracted. Individual patient-level consent was not required by the ethics review committees.

## Results

**Fig 1:**
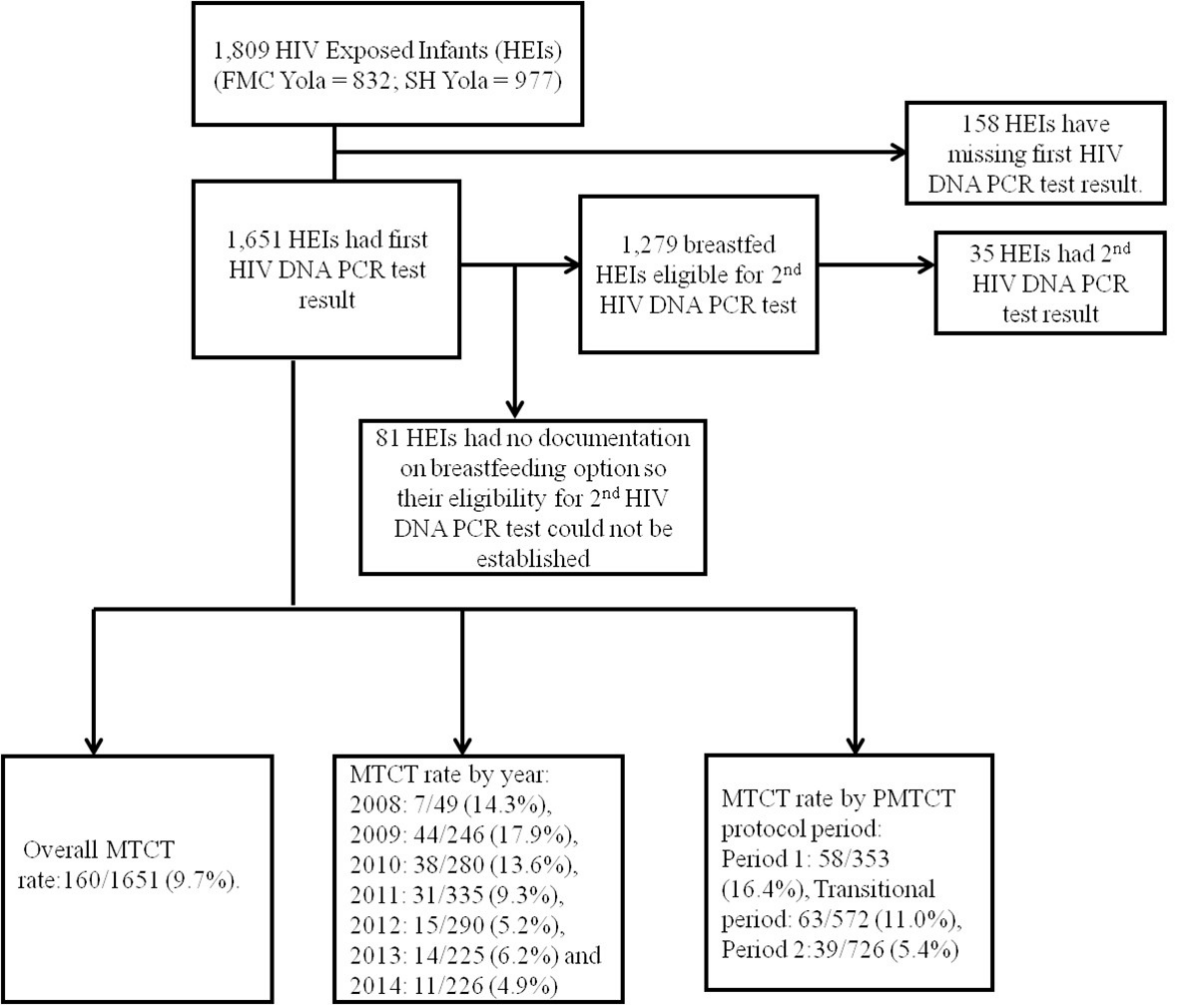
Flow chart for overall MTCT rate, MTCT rate by year and PMTCT protocol periods. Across the seven years under review, close to seventy percent (69.4%) of mothers of HEIs received either ART or ARVP; while approximately 66% of infants received some form of infant prophylaxis, as a single dose of Nevirapine (sdNVP), sdNVP with AZT, or daily NVP for 6 weeks. Of the 1,279 HEIs that were expected to have a second HIV DNA PCR test by virtue of their breastfeeding option (exclusively breastfed and mixed fed), only 35 (2.7%) of them had PCR results that could be longitudinally linked to the first result using routine data systems.

Data on 1,651 HEIs were included in the analyses (Fig 1). As shown in Table 1, more than half of the babies were males (51.3%); and only 30.2% of the babies were tested for HIV by the recommended age of 6 weeks. The commonest mode of feeding adopted by mothers at the time the infant was brought for HIV testing (median age of 8 weeks) was exclusive breastfeeding (53.9%); while mixed feeding (15.6%) was the least common for the entire 7 years under review.

**Table 1:** Baseline characteristics and prophylaxis status for HIV exposed infants (2008–2014) (N = 1,651).

Variable		FMC Yola	SH Yola	Total
		n (%)	n (%)	n (%)
**Gender**	Male	383 (51.3%)	453 (50.1%)	836 (50.6%)
	Female	363 (48.7%)	452 (49.9%)	815(49.4%)
	**Total**	746 (100.0%)	905 (100.0%)	1,651 (100.0%)
**Age**	≤6 weeks	276 (37.0%)	233 (24.6%)	499 (30.2%)
	>6 weeks to 2 months	148 (19.8%)	239 (26.4%)	387 (23.4%)
	>2months– 6 months	184 (24.7%)	257 (28.4%)	441 (26.7%)
	>6–12 months	106 (14.2%)	139 (15.4%)	245 (14.8%)
	>12 months	25 (3.4%)	34 (3.8%)	59 (3.6%)
	Missing	7 (0.9%)	13 (1.4%)	20 (1.2%)
	**Total**	746 (100.0%)	905 (100.0%)	1,651 (100.0%)
**Infant-Feeding Option**	Exclusive breastfeeding	381 (51.1%)	509 (56.2%)	890 (53.9%)
	Not breastfed, or Replacement feeding	252 (33.8%)	177 (19.6%)	429 (26.0%)
	Mixed feeding	70 (9.4%)	187 (20.7%)	257 (15.6%)
	Missing	43 (5.8%)	32 (3.5%)	75(4.5%)
	**Total**	746 (100.0%)	905 (100.0%)	1,651 (100.0%)
**Reason for PCR**	First test for healthy exposed infant	578 (77.5%)	707 (78.1%)	1,285 (77.8%)
	First test for sick infant	18 (2.4%)	12 (1.3%)	30 (1.8%)
	Problem with first test	150 (20.1%)	186 (20.6%)	336 (20.4%)
	**Total**	746 (100.0%)	905 (100.0%)	1,651 (100.0%)
**Maternal ARVs**	ART	417 (55.9%)	179 (19.8%)	596 (36.1%)
	ARVP	182 (24.4%)	177 (19.6%)	359 (21.7%)
	*ART or Triple Regimen	42 (5.6%)	149 (16.5%)	191 (11.6%)
	None	87 (11.7%)	130 (14.4%)	217 (13.1%)
	Missing	18 (2.4%)	270 (29.8%)	288 (17.4%)
	**Total**	746 (100.0%)	905 (100.0%)	1,651 (100.0%)
**Type of Maternal ARVP**	AZT + 3TC and sdNVP	134 (73.6%)	44 (24.9%)	178 (49.6%)
	AZT and sdNVP in labour	8 (4.4%)	27 (15.3%)	35 (9.7%)
	Triple regimen	35 (19.2%)	81 (45.8%)	116 (32.3%)
	sdNVP	3 (1.6%)	19 (10.7%)	22 (6.1%)
	Unknown	2 (1.1%)	1 (0.6%)	3 (0.8%)
	Missing	0 (0.0%)	5 (2.8%)	5 (1.4%)
	Total	182 (100.0%)	177 (100.0%)	359 (100.0%)
**Infant ARVs**	sdNVP at birth	35 (4.7%)	0 (0.0%)	35 (2.1%)
	sdNVP at birth and AZT for 4 weeks	252 (33.8%)	132 (14.6%)	384 (23.3%)
	NVP for 6 weeks	321 (43.0%)	346 (38.2%)	667 (40.4%)
	Unknown	3 (0.4%)	0 (0.0%)	3 (0.2%)
	None	109 (14.6%)	161 (17.8%)	270 (16.4%)
	Missing (ARV Type)	5 (0.7%)	4 (0.4%)	9 (0.5%)
	Missing (Prophylaxis use)	21 (2.8%)	262 (29.0%)	283 (17.1%)
	**Total**	746 (100.0%)	905 (100.0%)	1,651 (100.0%)

N = Total number of HIV-Exposed Infants

*Pregnant women took triple regimen, either solely for PMTCT (ARVP), or as ART (both for PMTCT and their health). Documentation was not strong enough to clearly delineate as ART, or ARVP.

### Overall MTCT rate and MTCT rate by year

The overall MTCT rate for all HEIs with first HIV DNA PCR test result, between 2008 and 2014 was 9.7% [160/1,651] (95% CI 8.3% - 11.1%) at a median age of 8 weeks (IQR = 6–20), with no statistically significant difference between the two sites. (95% CI of the rate difference = -4.4% - 1.2%; p = 0.273). Except for the increase seen between 2008–9 (from 14.3% [7/49] in 2008 to 17.9% [44/246] in 2009) and between and 2012–13 (from 5.2% [15/290] in 2012 to 6.2% [14/225] in 2013), the MTCT rate generally declined over the 7-year period (Table 2); and this decline was statistically significant (p = 0.016).

**Table 2:** Yearly trend of MTCT rate for FMC Yola and SH Yola at median age of 8 weeks (N = 1651).

	FMC Yola		SH Yola		Total	
Year	Number HIV positive (%)	95% CI of the %	Number HIV positive (%)	95% CI of the %	Number HIV positive (%)	95% CI of the %
2008	2 (14.3%)	*0.0%-33.3%	5 (14.3%)	2.5%-26.1%	7 (14.3%)	4.4%-24.2%
2009	14 (13.3%)	6.8%-19.9%	30 (21.3%)	14.5%-28.1%	44 (17.9%)	13.1%-22.7%
2010	16 (11.3%)	6%-16.5%	22 (15.9%)	9.8%-22.1%	38 (13.6%)	9.5%-17.6%
2011	17 (9.4%)	5.1%-13.7%	14 (9.1%)	4.5%-13.6%	31 (9.3%)	6.1%-12.4%
2012	4 (3.8%)	0.1%-7.4%	11 (6.0%)	2.5%-9.4%	15 (5.2%)	2.6%-7.7%
2013	5 (5.6%)	0.8%-10.3%	9 (6.7%)	2.4%-10.9%	14 (6.2%)	3.1%-9.4%
2014	8 (7.4%)	2.4%-12.4%	3 (2.5%)	*0.0%-5.4%	11 (4.9%)	2.1%-7.7%
Overall	66 (8.8%)	6.8%-10.9%	94 (10.4%)	8.4%-12.4%	160 (9.7%)	8.3%-11.1%

N = Total number of HIV Exposed Infants

* The confidence limit was amended, so that the confidence intervals were bounded between 0 and 100%.

### MTCT rate by PMTCT protocol periods

The lowest MTCT rate was in Period 2 (5.4% [39/726] (3.7%-7.0%)); while the highest was in Period 1 (16.4% [58/353] (12.6%-20.3%)), Table 3. The decline between Period 1 and Period 2 was statistically significant (p = 0.000).

**Table 3:** MTCT rate by periods of PMTCT protocol at median age of 8 weeks (N = 1651).

PMTCT Period	FMC Yola		SH Yola		Total	
	Number HIV positive (%)	95% CI of the %	Number HIV positive (%)	95% CI of the %	Number HIV positive (%)	95% CI of the %
Period 1 (Jan 2008 –Jan 2010)	20 (12.5%)	7.4%-17.6%	38 (19.7%)	14.1%-25.3%	58 (16.4%)	12.6%-20.3%
Transitional Period (Feb 2010 –May 2012)	30 (9.5%)	6.3%-12.7%	33 (12.8%)	8.7%-16.9%	63 (11.0%)	8.4%-13.6%
Period 2 (Jun 2012-Dec 2014)	16 (5.9%)	3.1%-8.7%	23 (5.1%)	3.0%-7.1%	39 (5.4%)	3.7%-7.0%
Total	66 (8.8%)	6.8%-10.9%	94 (10.4%)	8.4%-12.4%	160 (9.7%)	8.3%-11.1%

*Period 1 PMTCT regimen*: Mother: Ante partum—AZT from 28 weeks of gestation or AZT+3TC from 34–36 weeks of gestation; Intrapartum—SdNVP +AZT+3TC at onset of labour; Postpartum—AZT+3TC for 7 days Infants: sdNVP at birth (preferably within 72 hours) plus AZT for 6 weeks. *Period 2 PMTCT regimen*: Mother: AZT+ 3TC + (EFV or NVP or LPV/r) or TDF+ 3TC+ EFV from 14 weeks of gestation up to 1-week post cessation of breastfeeding. Infant: NVP at birth (preferably within 72 hours) up to 6 weeks of age. *Transitional Period*: Both Period 1 and Period 2 Regimens (both regimens were in use during this period, due to delayed transition).

### Predictors of MTCT

Multiple regression analysis revealed that infant age, breastfeeding option, antiretroviral regimen and year predicted MTCT. HEIs older than 12 months had 3.3 times (adjusted odds ratio (AOR)), the odds of being HIV positive than those ≤6 weeks; while mixed fed infants had 2.4 times the odds of being HIV positive than infants that were not breastfed. The odds of infant HIV positivity was also higher (AOR = 26.4), when neither the mother, nor the infant received prophylaxis. HEIs born in 2012 had lower odds of being HIV positive than those born in 2008 (Table 4).

**Table 4:** Multivariable analysis of the factors associated with MTCT at median age of 8 weeks, using the cumulative pooled data.

Variable		Crude Odds Ratio	95% CI	P value	Adjusted Odds Ratio	95% CI	P value
Gender	Male	1.0			1.0		
	Female	1.3	1.0–1.8	0.096	1.2	0.7–1.9	0.546
^+^Infant Age	≤6 weeks	1.0			1.0		
	>6 weeks—2 months	1.1	0.5–2.4	0.766	0.8	0.3–2.1	0.636
	>2–6 months	4.0	2.2–7.3	0.000	1.7	0.8–3.7	0.198
	>6–12 months	8.7	4.8–15.8	0.000	1.8	0.8–4.1	0.194
	>12 months	25.5	12.6–51.4	0.000	*3.3	**1.2–8.8**	0.017
^++^Breastfeeding option	Not Breast-fed	1.0			1.0		
	Exclusively Breast-fed	1.6	1.0–2.6	0.056	1.9	1.0–3.9	0.058
	Mixed fed	5.0	3.0–8.4	0.000	*2.4	**1.1–5.5**	0.032
ART/ARV Prophylaxis Receipt	Both Mother and Infant	1.0			1.0		
	Mother alone	3.3	1.4–7.7	0.008	*2.8	**1.1–7.0**	0.028
	Infant alone	4.3	1.4–12.8	0.010	*5.6	**1.8–17.8**	0.003
	Neither Mother, nor Infant	38.5	23.4–63.4	0.000	*26.4	**14.0–49.8**	0.000
Hospital	FMC Yola	1.0			1.0		
	SH Yola	1.2	0.9–1.7	0.293	0.5	0.3–0.8	0.005
Year	2008	1.0			1.0		
	2009	1.3	0.6–3.1	0.544	1.2	0.3–5.6	0.794
	2010	0.9	0.4–2.3	0.893	0.5	0.1–2.1	0.327
	2011	0.6	0.3–1.5	0.275	0.3	0.1–1.5	0.154
	2012	0.3	0.1–0.9	0.022	*0.2	**0.0–1.0**	**0.049**
	2013	0.4	0.2–1.1	0.062	0.3	0.1–1.6	0.169
	2014	0.3	0.1–0.8	0.021	0.2	0.1–1.3	0.097

N = 1,278; Pseudo R^2^: 37.3%

*Statistically significant odds ratio using pooled data from the two health facilities

^+^Proportions of mother infant-pair that did not receive ARVP/T were 53.7% and 2.1% for HEIs > 12 months and those ≤6 weeks, respectively–analysis restricted to mother-infant pair with information on ARV use for both mother and infant

^++^Proportions of mother-infant pair that did not receive ARVP/T were 37.5%, 10.3% and 4.9% for mixed fed, exclusively breast-fed and HEIs that were not breast-fed, respectively.

## Discussion

Using routine data (2008–2014) from two health facilities, we demonstrated a decline in MTCT rate with improvements in the PMTCT protocol. Overall, the MTCT rate was 9.7% with a declining trend from 14.3% in 2008, to 4.9% in 2014 (p = 0.016). The MTCT rate was lowest (5.4%) during the period when Option B was in use for ARVT/P. Infant age, breastfeeding option, antiretroviral regimen and year were significant risk factors for MTCT.

The MTCT rate of 22.0% and 22.5% from studies conducted by Afe et al., (2011) and Audu et al. (2014) using data gathered between February 2007 and October 2008 at six health facilities (each) in Lagos State, Nigeria fell within our confidence intervals for 2008 (14.3% (95%CI 4.4% - 24.2%)) [19,20]. Our overall MTCT rate estimate of 11.25%, using pooled data from 2008–2012 was very close to the MTCT rate of 11.0% at 17 weeks, found in four secondary health facilities in Kwara and Niger States, Nigeria, 2009–2012 [30]. It was also close to the MTCT rate of 9.8% (mean age = 17.83 weeks) reported by Inalegwu et al. (2016), who analysed data of the first HIV DNA PCR test results from 150 health facilities, 2008–2012 [31]. Our point estimate of 4.9% in 2014 is lower than the 6.0% reported by Khamofu et al. (2015), and 13.0% at 6 weeks, projected for Nigeria by UNAIDS [6, 32]. Modelled data suggest that between 2009 and 2015, new HIV infections among children in Nigeria declined by 21% [32]. This progress is, however, judged to be slow; as Nigeria and Angola are the only two countries with less than 33% reduction in new HIV infections among children [32].

We observed a general decline in MTCT rate, with the introduction of more efficacious regimen [3,11,12] with the lowest MTCT rate of 5.4% during the period when the triple regimen was in use for ARVT/P. We also noted lower odds of being HIV positive in 2012 compared to 2008 in the adjusted analysis. These findings further buttressed the need for countries to adopt either Option B or Option B^+^ as the preferred PMTCT regimen. While it is commendable that Nigeria adopted Option B+ in June 2016 [32] the country will only be able to achieve virtual elimination of MTCT (reduction of MTCT risk to <5%) if the coverage of ARVT/P, which stood at 30% by 2016, can be improved [6, 32]. Additionally, intensified training and monitoring is required to ensure prompt migration to the new protocol, given that prescribing habit would not necessarily change immediately. The country would also need to implement strategies to ensure adherence as there are concerns that asymptomatic pregnant women living with HIV, who do not necessarily require ART for their health, could discontinue treatment; and they would be ultimately lost to follow up [33,34]. A study conducted in Haiti reported higher attrition among pregnant women living with HIV initiated on ART under Option B+ compared to non-pregnant women on ART; while a readiness assessment conducted by Erekaha et al. in North-Central Nigeria suggested that some pregnant women living with HIV would only take medication to protect their babies; and they might well stop after delivery [33, 34].

As reported in other studies conducted in Africa, not all mother-infant pairs in our study received antiretroviral drugs, despite the availability of these interventions [35,36]. Measures must therefore be implemented to improve the utilization of PMTCT services.

The higher odds of MTCT among infants older than12 months could be attributed to post-natal MTCT because of prolonged breastfeeding. A higher proportion of mother-infant pairs in this age bracket, when compared to those ≤6 weeks of age, also did not receive antiretroviral drugs. Our study demonstrates that mixed-fed infants have significantly higher odds of being HIV positive than formula-fed infants. This could be linked to the lack of ARV prophylaxis cover for a higher proportion of the mixed-fed infants. Mixed feeding is known to disrupt the infant’s intestinal lining, thereby increasing the chances of HIV transmission, especially when there is poor ART coverage, with consequent poor maternal viral load suppression [37]. The adoption of mixed-feeding practice in settings with poor ART coverage and adherence would, therefore, be risky [38].

Overall, our study provided evidence for the effectiveness of PMTCT in service delivery settings, with complexities that are not accounted for by controlled conditions under which efficacy studies are conducted [39–42].

Our study has several limitations: 1) We only reviewed records of HEIs brought by their caregivers for HIV DNA PCR testing, and for whom results were available; the exclusion of non-attendees may have under-estimated the MTCT rate 2). We also did not assess the effect of mode of delivery, maternal CD4+ cell count, viral load, duration of ARVT/P use and adherence on the MTCT rate. Finally, given that the second HIV DNA PCR test results were only available for 2.7% of the HEIs, our estimates largely assessed MTCT rate at the time of first testing, and not at the final end-point (post-cessation of breastfeeding). However, the strengths of our study lie in: 1) the extensive number of years reviewed. This allowed us to provide evidence on PMTCT effectiveness during two distinct periods of PMTCT protocol implementation, as well as in the transitional period in Nigeria; 2) inclusion of a health facility that provides predominantly secondary-level healthcare services; this level has less specialized health personnel, compared with tertiary or teaching hospitals; 3) our large sample sizes, which were higher than most of the other Nigerian studies.

## Conclusion

Our MTCT rate estimates demonstrated the effectiveness of PMTCT interventions in routine settings, as the PMTCT regimens became more intense with earlier initiation of triple therapy. Thus, efforts to scale up lifelong ARVT/P (Option B+) in Nigeria should be accelerated. Given the increased coverage of PMTCT services over the past 16 years, it is important that Nigeria carries out a survey-based population-level evaluation of her PMTCT program, as is done in other countries, like South Africa. This could help in assessing the progress towards Nigeria’s PMTCT targets [3].

## Supporting information

S1 Table ART and PMTCT eligibility criteria and regimens in Nigeria. (DOCX)

S2 Table Comparison of Baseline characteristics and prophylaxis status for HIV exposed infants with and without first HIV DNA PCR test results. (DOCX)

S1 Fig Flow chart showing the recommended cascade of PMTCT interventions in Nigeria, 2008–2014. (TIF)

S1 File Study data. (XLSX)
