# Correction: Trends and predictors of mother-to-child transmission of HIV in an era of protocol changes: Findings from two large health facilities in North East Nigeria

**DOI:** 10.1371/journal.pone.0226665

**Published:** 2019-12-12

**Authors:** The PLOS ONE Staff

The second and third paragraphs of the Results section incorrectly appear in the Fig 1 caption. The second paragraph of the Results section is: “Across the seven years under review, close to seventy percent (69.4%) of mothers of HEIs received either ART or ARVP; while approximately 66% of infants received some form of infant prophylaxis, as a single dose of Nevirapine (sdNVP), sdNVP with AZT, or daily NVP for 6 weeks.” The third paragraph of the Results section is: “Of the 1,279 HEIs that were expected to have a second HIV DNA PCR test by virtue of their breastfeeding option (exclusively breastfed and mixed fed), only 35 (2.7%) of them had PCR results that could be longitudinally linked to the first result using routine data systems.” The publisher apologizes for the errors.

**Fig 1:**
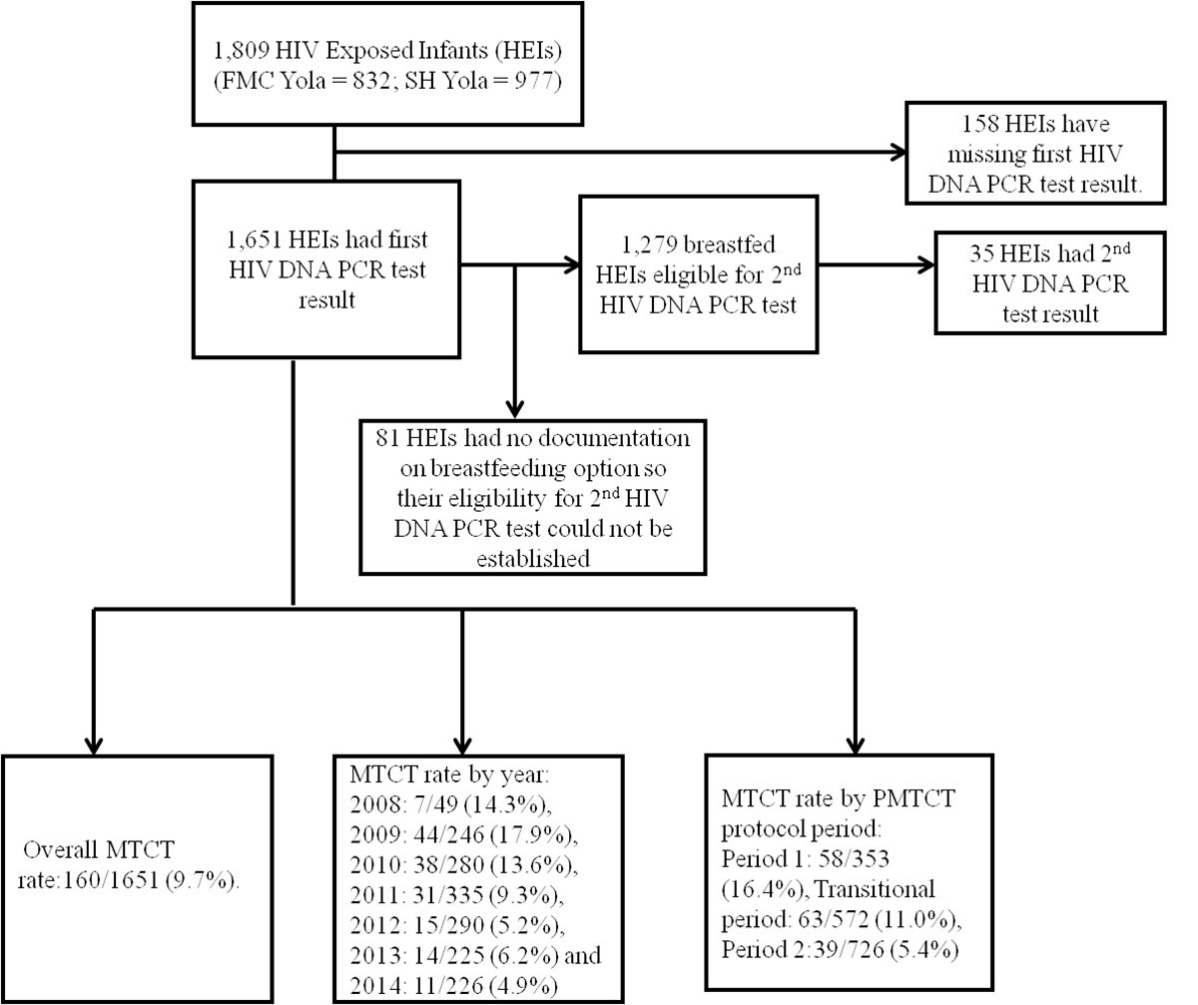
Flow chart for overall MTCT rate, MTCT rate by year and PMTCT protocol periods.

Please see the complete, correct Fig 1 and Fig 1 caption here.
