# Supplementary material for: Trends and predictors of mother-to-child transmission of HIV in an era of protocol changes: Findings from two large health facilities in North East Nigeria
Source: PLoS One. 2019 Nov 11;14(11):e0224670. doi: 10.1371/journal.pone.0224670 (PMC6844480; doi:10.1371/journal.pone.0224670)
Supplement: S1 Table — (DOCX) [file pone.0224670.s001.docx]

**S1 Table: ART and PMTCT eligibility criteria and regimens in Nigeria, 2007-2014**

|  | **2007 National Guidelines** | **2010 National Guidelines** | | **2014 National Guidelines** |
| --- | --- | --- | --- | --- |
|  | **PMTCT Eligibility Criteria/PMTCT Regimens** | | | |
| Mother | WHO Clinical Stage I, II with CD4+ cell count >200 cells/mm^3^; WHO Clinical Stage III with CD4^+^ cell count >350 cells/mm^3^  *Antepartum*  Give AZT from 28 weeks of gestation or AZT+3TC from 34-36 weeks of gestation  *Intrapartum*  SdNVP +AZT+3TC at onset of labour  *Postpartum*  AZT+3TC for 7 days | Health Facility CAN monitor triple ARV (*Option B*) | **Health Facility CANNOT monitor triple ARV (*Option A*)** | WHO Clinical Stage I or II with CD4^+^ cell count >500 cells/mm^3^  Give TDF/3TC/EFV at the time of diagnosis up to 1-week post-cessation of breast-feeding |
|  |  | WHO Clinical Stage I or II with CD4^+^ cell count >350 cells/mm^3,^  Give AZT+ 3TC +EFV or NVP or LPV/r;  TDF+ 3TC+ EFV from 14 weeks of gestation up to 1-week post cessation of breast-feeding | WHO Clinical Stage I or II with CD4^+^ cell count >350 cells/mm^3^  Give AZT from 14 weeks gestation, sdNVP at onset of labour and delivery, AZT+3TC 12hourly during labour and delivery and 12 hourly for 7 days post-partum |  |
| *Infant | sdNVP at birth (preferably within 72 hours) plus AZT for 6 weeks. | NVP at birth (preferably within 72 hours) up to 6 weeks of age. | *Not Breast-feeding*  NVP at birth (preferably within 72 hours) up to 6 weeks of age  *Breast-feeding*  NVP at birth (preferably within 72 hours) up to 1-week post cessation of breastfeeding | NVP at birth (preferably within 72 hours) up to 6 weeks of age. |
| **ART Eligibility Criteria/ART Regimens** | | | | |
| Mother | WHO Clinical Stage IV irrespective of CD4^+^ cell count  WHO Clinical Stage III if CD4^+^ cell count < 350 cells/mm^3^  WHO Clinical Stage I and II if CD4^+^ cell count is ≤ 200 cells/mm^3^  Give AZT+ 3TC +EFV or NVP or LPV/r | WHO clinical stages III or IV irrespective of CD4^+^ cell count and CD4+ cell count ≤350 cells/mm^3^ irrespective of WHO clinical staging  Give AZT+3TC+LPV/r or EFV or ABC; TDF+3TC or FTC + EFV | WHO clinical stages III or IV irrespective of CD4^+^ cell count and CD4+ cell count ≤350 cells/mm3 irrespective of WHO clinical staging  Give AZT+3TC+LPV/r or EFV or ABC; TDF+3TC or FTC + EFV | WHO clinical stages III or IV irrespective of CD4+ cell count and CD4+ cell count ≤500 cells/mm3 irrespective of WHO clinical staging  Give TDF/3TC/EFV |
| Infant | WHO pediatric HIV Clinical Stage III or IV irrespective of CD4^+^%  WHO pediatric HIV Clinical Stage II if CD4^+^% < 20%  Give AZT+3TC+NVP or EFV. ABC+3TC+NVP or EFV d4T+3TC+NVP or EFV | All infants ≤ 2 years    Give AZT+3TC+NVP or LPV/r or ABC or EFV. ABC+3TC+NVP or LPV/r  d4T+3TC+NVP or LPV/r | All infants ≤ 2 years  Give AZT+3TC+NVP or LPV/r or ABC or EFV. ABC+3TC+NVP or LPV/r d4T+3TC+NVP or LPV/r | All infants ≤5 years  Give AZT+3TC+NVP or LPV/r or ABC or EFV |

3TC, Lamivudine; ABC, Abacavir; ARV, Antiretroviral drug; AZT, Zidovudine; CTX, Co-trimoxazole; FTC, Emtricitabine; LPV/r, Lopinavir/ritonavir; NVP, Nevirapine; sdNVP, single dose; Stavudine, d4T; Tenofovir, TDF; WHO, World Health Organization. *Initiate CTX at 6 weeks and continue until infant is confirmed to be HIV negative post-cessation of breast-feeding

NB: SH Yola and FMC Yola commenced implementation of 2007 National guidelines in September 2007 and January 2008, respectively; while the implementation of Option B of 2010 National Guidelines commenced in February 2010 at the two health facilities
