# Supplementary material for: Trends and predictors of mother-to-child transmission of HIV in an era of protocol changes: Findings from two large health facilities in North East Nigeria
Source: PLoS One. 2019 Nov 11;14(11):e0224670. doi: 10.1371/journal.pone.0224670 (PMC6844480; doi:10.1371/journal.pone.0224670)
Supplement: S2 Table — (DOCX) [file pone.0224670.s002.docx]

**S2 Table: Comparison of Baseline characteristics and prophylaxis status for HIV exposed infants with and without first HIV DNA PCR test results**

| **Variable** | | **HEIs with HIV DNA PCR Test Result** | **HEIs without HIV DNA PCR Test Result** | **Total** | **P-value** |
| --- | --- | --- | --- | --- | --- |
|  |  | **n (%)** | **n (%)** | **n (%)** |  |
| **Gender** | Male | 836 (50.6%) | 79 (50.0%) | 915 (50.6%) | 0.879 |
|  | Female | 815(49.4%) | 79 (50.0%) | 894(49.4%) |  |
|  | **Total** | 1,651 (100.0%) | 158 (100.0%) | 1,809 (100.0%) |  |
| **Age** | ≤6 weeks | 499 (30.2%) | 44 (27.8%) | 543 (30.0%) | **0.001** |
|  | >6 weeks to 2 months | 387 (23.4%) | 28 (17.7%) | 415 (22.9%) |  |
|  | >2months – 6 months | 441 (26.7%) | 39 (24.7%) | 480 (26.5%) |  |
|  | >6 - 12 months | 245 (14.8%) | 28 (17.7%) | 273 (15.1%) |  |
|  | >12 months | 59 (3.6%) | 11 (7.0%) | 70 (3.9%) |  |
|  | Missing | 20 (1.2%) | 8 (5.1%) | 28 (1.5%) |  |
|  | **Total** | 1,651 (100.0%) | 158 (100.0%) | 1,809 (100.0%) |  |
| **Infant Feeding Option** | Exclusive breast-feeding | 890 (53.9%) | 100 (63.3%) | 990 (54.7%) | **0.002** |
|  | Not breast-fed or  Replacement feeding | 429 (26.0%) | 20 (12.7%) | 449 (24.8%) |  |
|  | Mixed feeding | 257 (15.6%) | 32 (20.2%) | 289 (16.0%) |  |
|  | Missing | 75(4.5%) | 6 (3.8%) | 81 (4.5%) |  |
|  | **Total** | 1,651 (100.0%) | 158 (100.0%) | 1,809 (100.0%) |  |
| **Reason for PCR** | First test for healthy exposed infant | 1,285 (77.8%) | 118 (74.7%) | 1,403 (77.6%) | 0.500 |
|  | First test for sick infant | 30 (1.8%) | 2 (1.3%) | 32 (1.8%) |  |
|  | Problem with first test | 336 (20.4%) | 38 (24.0%) | 374 (20.7%) |  |
|  | **Total** | 1,651 (100.0%) | 158 (100.0%) | 1,809 (100.0%) |  |
| **Maternal ARVs** | ART | 596 (36.1%) | 8 (5.0%) | 604 (33.3%) | **0.000** |
|  | ARVP | 359 (21.7%) | 11 (7.0%) | 370 (20.4%) |  |
|  | *ART or Triple Regimen | 191 (11.6%) | 75 (47.5%) | 266 (14.7%) |  |
|  | None | 217 (13.1%) | 14 (8.9%) | 231 (12.8%) |  |
|  | Missing | 288 (17.4%) | 50 (31.6%) | 338 (18.7%) |  |
|  | **Total** | 1,651 (100.0%) | 158 (100.0%) | 1,809 (100.0%) |  |
| **Type of Maternal ARVP** | AZT + 3TC and sdNVP | 178 (49.6%) | 2 (18.2%) | 180 (48.7%) | 0.154 |
|  | AZT and sdNVP in labour | 35 (9.7%) | 3 (27.3%) | 38 (10.3%) |  |
|  | Triple regimen | 116 (32.3%) | 4 (36.3%) | 120 (32.4%) |  |
|  | sdNVP | 22 (6.1%) | 2 (18.2%) | 24 (6.5%) |  |
|  | Unknown | 3 (0.8%) | 0 (0.0%) | 3 (0.8%) |  |
|  | Missing | 5 (1.4%) | 0 (0.0%) | 5 (1.4%) |  |
|  | Total | 359 (100.0%) | 11 (100.0%) | 370 (100.0%) |  |
| **Infant ARVs** | sdNVP at birth | 35 (2.1%) | 1 (0.6%) | 36 (2.0%) | **0.000** |
|  | sdNVP at birth and AZT for 4 weeks | 384 (23.3%) | 3 (1.9%) | 387 (21.4%) |  |
|  | NVP for 6 weeks | 667 (40.4%) | 93 (58.9%) | 760 (42.0%) |  |
|  | Unknown | 3 (0.2%) | 0 (0.0%) | 3 (0.2%) |  |
|  | None | 270 (16.4%) | 19 (12.0%) | 289 (16.0%) |  |
|  | Missing (ARV Type) | 9 (0.5%) | 0 (0.0%) | 9 (0.5%) |  |
|  | Missing (Prophylaxis use) | 283 (17.1%) | 42 (26.6%) | 325 (18.0%) |  |
|  | **Total** | 1,651 (100.0%) | 158 (100.0%) | 1,809 (100.0%) |  |
