# Supplementary figures and images for: Trends and predictors of mother-to-child transmission of HIV in an era of protocol changes: Findings from two large health facilities in North East Nigeria
Source: PLoS One. 2019 Nov 11;14(11):e0224670. doi: 10.1371/journal.pone.0224670 (PMC6844480; doi:10.1371/journal.pone.0224670)

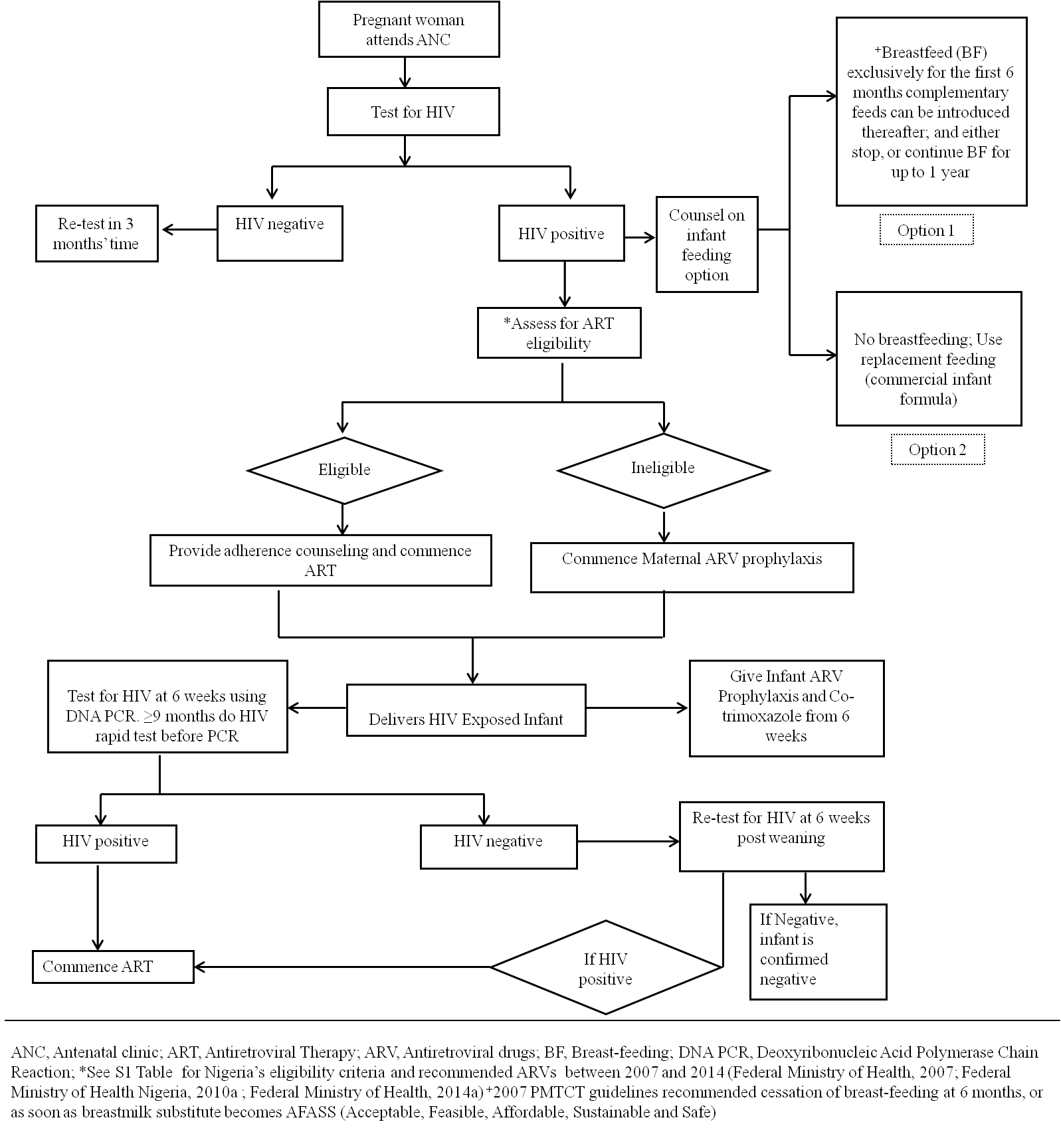

Supplement: S1 Fig — (TIF) [file pone.0224670.s003.tif]
